# RETRACTION: HDAC‐Inhibitor (S)‐8 Disrupts HDAC6‐PP1 Complex Prompting A375 Melanoma Cell Growth Arrest and Apoptosis

**DOI:** 10.1111/jcmm.71046

**Published:** 2026-01-29

## Abstract

**RETRACTION**: BalliuM. , GuandaliniL. , RomanelliM.N. , D'AmicoM. and PaolettiF. , “HDAC‐Inhibitor (S)‐8 Disrupts HDAC6‐PP1 Complex Prompting A375 Melanoma Cell Growth Arrest and Apoptosis,” Journal of Cellular and Molecular Medicine 19, no. 1 (2015): 143–154, 10.1111/jcmm.12345.25376115 PMC4288358

The above article, published online on 06 November 2014 in Wiley Online Library (wileyonlinelibrary.com), has been retracted by agreement between the journal Editor‐in‐Chief, Stefan N. Constantinescu; the Foundation for Cellular and Molecular Medicine; and John Wiley & Sons Ltd. The retraction has been agreed upon following an investigation into concerns raised by a third party. The investigation identified duplication involving α‐tubulin bands in Figure 3A and AKT bands in Figure 3D. Furthermore, the GAPDH bands presented in Figure 7C appear to be duplicated in another article published later by two of the same authors. The authors were invited to comment on the concerns and provide supporting data. While the authors cooperated with the investigation and provided some data, the data did not correspond to the published images. Given the nature of the concerns, the editors have lost confidence in the results and conclusions. The authors disagree with the retraction.

**RETRACTION**: M. Balliu , L. Guandalini , M.N. Romanelli , M. D'Amico and F. Paoletti , “HDAC‐Inhibitor (S)‐8 Disrupts HDAC6‐PP1 Complex Prompting A375 Melanoma Cell Growth Arrest and Apoptosis,” Journal of Cellular and Molecular Medicine 19, no. 1 (2015): 143–154, 10.1111/jcmm.12345.25376115 PMC4288358

The above article, published online on 06 November 2014 in Wiley Online Library (wileyonlinelibrary.com), has been retracted by agreement between the journal Editor‐in‐Chief, Stefan N. Constantinescu; the Foundation for Cellular and Molecular Medicine; and John Wiley & Sons Ltd. The retraction has been agreed upon following an investigation into concerns raised by a third party. The investigation identified duplication involving α‐tubulin bands in Figure 3A and AKT bands in Figure 3D. Furthermore, the GAPDH bands presented in Figure 7C appear to be duplicated in another article published later by two of the same authors. The authors were invited to comment on the concerns and provide supporting data. While the authors cooperated with the investigation and provided some data, the data did not correspond to the published images. Given the nature of the concerns, the editors have lost confidence in the results and conclusions. The authors disagree with the retraction.

